# 

**Published:** 1878-10-20

**Authors:** 


ORIGINAL AND SELECTED ARTICLES.

Remarks on the cause and treatment of Yellow Fever and the Bedford (Va.) Springs Alum and Iron Mass as a Remedial Agent —By W. A. Greene, M. D., Macon. Ga., Ex-President Georgia Medical Association ; formerly chief Surg. of Artillery, 3rd Army Corps, A N. Va.......289.

Yellow Fever ; by T. B. Greenley, M D., of Ky.......295

Cholagogues.......298

Fever of Dentition.......300

Treatment of Typhoid Fever.......300

Cauterization of Cervix in Vomiting of Pregnancy ; By John M. Boring, M. D , Atlanta, Ga.......302

Treatment of Uterine Fibroids with Galvanism by Profound Puncture.......303

ABSTRACTS AND GLEANINGS.

Proceedings in French Societies. Porro's Operation, Another Successful Case. 304 ; Tinc. Iodine in Fevers, 305; The Ambulatory Treatment of Psoriasis Vulgaris. Recent Progress in Pathological Anatomy 306 ; Aids to Labor. 307 ; Ether and Chloroform in Puerperal convulsions. Bryonia and Drasera in the Treatment of Whooping-Cough. 309 ; Tapping a Vomica in Phthisis. A Vehicle for Quinine, Treatment of Mammary Abscess, 310 ; Proceedings in French societies. Cæsaraen Section After Death. Oxalate of Cerium and Caffein as Preventatives of the Nauseating effects of opium.—By Samuel C. Busey, M. D., 311 ; Vinegar in Post Partum Hemmorreage. 312 ; Arsenic and its Preparations in the Treatment of Skin Diseases. Chronic chills, 313.

NOTES, QUERIES AND FORMULÆ.

Felons, Smartweed, 314 ; Glycerite of the Hypophosite of Zinc. Antidote to carbolic Acid. Iodine In Intermittents. common Salt as a Remedy for Intermittent Fever, 315 ; Antidote to Arsenic. chlorodyne (G. H. F. Toledo, Ohio.) Postage Stamp Mucilage (Hawkins Tex.) To remove Foreign Bodies from the Nose. Pulvis Paregoricus. Tonic in Anæmia. 316

SCIENTIFIC ITEMS.

Photographing with Lightning. The Moon' An Electric Awakener. The Polymicroscope, 317.

EDITORIAL AND MISCELLANEOUS.

Cinchonia Alkaloid. Martyred Pyhsicians. 318 ; True Bravery. Elementary Quantitative Analysis, by A. Classen, Berberis Aquifolium. Antagonism of Theraapeutic Agents and What it Teaches. The Physicians Visiting List for 1878, 319 ; Yellow Fever. Aloe & Hernstein. Liberal Offers. Pharmacental Association, Apologetic. Milk Diet in the Perpeural State Surgeon General's Fever Circular. Receipted. Notice, 320.

